# Validating a rapid psychophysical procedure for estimating interaural time difference thresholds

**DOI:** 10.1016/j.heares.2025.109491

**Published:** 2025-11-26

**Authors:** Justin M. Aronoff, Jordan Deutsch, Josephine R. LaPapa, Karla Rodriguez, Leslie R. Bernstein

**Affiliations:** a University of Illinois at Urbana-Champaign, 901 S 6th St., Champaign, IL 61820, USA; b University of Connecticut, 263 Farmington Avenue, Farmington, CT 06030-3401, USA

**Keywords:** Interaural time differences, Psychophysical procedures, Descending series procedure, Adaptive procedure

## Abstract

The detection and discrimination of interaural time differences (ITDs) underpin many binaural abilities. Obtaining precise “threshold”-ITDs is, however, often quite time consuming. This study investigated the use of a rapid, descending series procedure to estimate threshold-ITDs. The procedure involves beginning with a suprathreshold ITD and then decreasing the ITD over sequential trials independent of response accuracy, ending with subthreshold ITDs. Normal hearing participants were tested using both an adaptive and descending series procedure. The results from the two procedures were comparable, while the descending series procedure required approximately half the time to complete as did the adaptive procedure. A function was determined for estimating ITD thresholds from the number of correct responses obtained with the descending series procedure. The results indicate that a descending series procedure is an efficient approach for estimating threshold-ITDs.

## 1. Introduction

Interaural time differences (ITDs) are a fundamental cue underlying binaural abilities. Current approaches to obtaining “threshold”^1^ -ITDs often employ the method of constant stimuli or adaptive procedures (e. g., Bernstein et al., 2001; Hu and Dietz, 2015; Staisloff and Aronoff, 2021; Thavam and Dietz, 2019). The method of constant stimuli, when used to estimate threshold-ITDs, uses a fixed set of ITDs ordered randomly, each one being visited multiple times. While this method affords the calculation of a psychometric function, it is one of the most time-consuming procedures for estimating thresholds. An alternative approach is an adaptive procedure, which involves adjusting the magnitude of ITD either up or down, contingent on a participant’s responses and according to pre-determined rules (Levitt, 1971). This type of testing follows an iterative process that continues until a fixed number of “reversals” (i.e. trials where ITDs change from increasing to decreasing or from decreasing to increasing) are achieved. Adaptive procedures can be quicker than the method of constant stimuli because most of the trials are presented at ITDs near threshold. Adaptive procedures, however, can still be time consuming, even if less so than the method of constant stimuli.

An alternative approach is a descending series procedure in which trials begin at a suprathreshold value of ITD with ITD-magnitude decreased sequentially across trials, regardless of a participant’s responses, ending with subthreshold ITDs. A percent correct score is then calculated across all trials. The descending procedure has been used previously to estimate thresholds for spatial release from masking (Gallun et al., 2013) and speech recognition in noise (McArdle et al., 2005), and is amenable to ITD testing. Additionally, there are variants of this approach, such as one that uses multiple short descending tracks, adapting the stimulus parameter range and starting value based on previous tracks (e.g., Lelo de Larrea-Mancera et al., 2023). The goals of this study were to assess whether both the adaptive and descending series procedures yield comparable results and whether the resulting percent correct score can be used to calculate the threshold-ITD for a given level of performance that matches the threshold-ITD obtained with an adaptive procedure targeting that same level of performance. If so, then because the descending procedure typically requires only one to two trials at each ITD (Gallun et al., 2013), it would be more rapid than adaptive procedures while maintaining similar accuracy.

## 2. Methods

### 2.1. Participants

Eighteen normal hearing listeners participated in this experiment (six males). Their ages ranged from 18 to 56 years; the median age was 21 years. All had pure tone thresholds ≤ 25 dB HL from 250 to 8 kHz and no more than a 10 dB difference in thresholds across ears for any frequency tested.

### 2.2. Stimuli

**Fig. 1.**
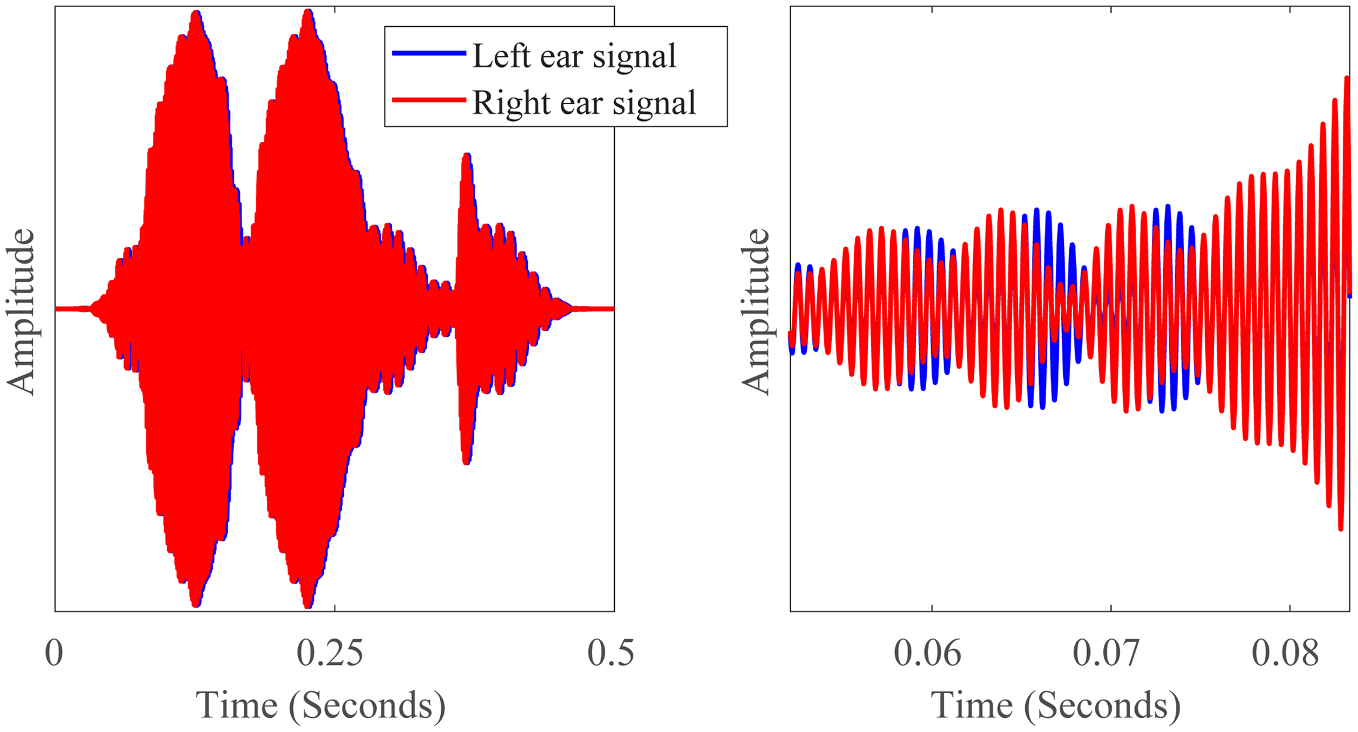
Example stimulus with a 1000-μs ITD. The right panel shows a small section of the left panel time-waveform.

The stimuli consisted of a 500-ms-long, 1500-Hz pure tone carrier modulated by envelope functions derived from AzBio (Spahr et al., 2012) sentences, similar to those used in Aronoff et al. (2025). Those envelope functions were drawn from a single channel of a 22-channel vocoder. Thus, the modulators were narrowband speech envelopes. The envelopes were aperiodic and were not identifiable as speech (see Fig. 1). Raised cosine ramps, 100-ms in duration, were applied to the onsets and offsets of the stimuli. ITDs were implemented by shifting the entire waveform in the time domain after application of the ramps. The stimuli were generated at a sampling rate of 96 kHz.

### 2.3. Procedures

The stimuli were presented using an M-Audio MobilePre external soundcard and were delivered over Sennheiser HDA 200 headphones. The left and right headphones were calibrated separately using an artificial ear, microphone, and preamplifier (Brüel and Kjær type 4153, 4966, and 2245, respectively). Stimuli were presented at 60 dBA.

A four-interval, two-alternative forced-choice task was used. The same randomly selected envelope was used for both ears and for all four stimuli within a trial, with the exception that an ITD was added to one of the stimuli. Participants viewed a screen with four buttons arranged vertically and labeled from the top, “First”, “Second”, “Third”, and “Fourth,” corresponding to the four observation intervals within each trial. Stimuli were presented sequentially with an inter-stimulus interval of 500 to 600 ms, with the specific duration within that range chosen randomly. The first and last interval contained diotic stimuli and served as references. With equal probability, either the second or third interval contained a stimulus with a non-zero ITD. The ITD always led in the right ear. The first and last buttons were grayed out and could not be selected as responses. Participants were instructed to click the button corresponding to the interval containing the stimulus that differed from the other three. Participants were instructed that the “target” stimulus might be perceived “off to the side.” The next trial started after the participant responded. The order of completion of the adaptive or descending series was determined randomly for each participant.

#### 2.3.1. Descending series procedure

The initial ITD was 1000 μs. Participants completed two trials at that ITD, after which the ITD was decreased by 2 dB (i.e., to 631 μs)^2^ and two trials were presented at the reduced ITD. This process was repeated until participants completed two trials with an ITD of 16 μs. There were twenty trials in total, with two trials for each of ten different ITDs. Participants completed five blocks of the descending series procedure.

#### 2.3.2. Adaptive procedure

The initial ITD was 1000 μs. ITDs were adjusted using a two down-one up adaptive rule with a 2-dB step-size, yielding estimates of threshold-ITD corresponding to 70.7% correct (Levitt, 1971). The run continued until 10 reversals occurred. For each participant, an estimate of threshold was calculated as the geometric mean of the ITDs corresponding to the last six reversals. Participants completed five blocks of the adaptive procedure.

## 3. Results

Robust statistics were used because there is ample evidence that they, typically, yield more valid measurements and better power than traditional approaches (Erceg-Hurn and Mirosevich, 2008; Wilcox, 1998; Wilcox and Keselman, 2003). These include methods such as trimmed means, specifically, the mean of the central 60% of the data, bootstrap analyses, in which random sampling with replacement is used to analyze distributions reflecting those in the original data rather than normal distributions, and least trimmed squares regressions, a robust regression method less affected by “outlier” data points than traditional least squares regression.

Two participants were excluded because their adaptive procedure threshold-ITD was greater than 1000 μs, the starting point of the descending series procedure. As a result, data from sixteen participants were analyzed. The 20% trimmed mean of the adaptive procedure thresholds and the percent correct pooled across runs for each ITD used for the descending series were calculated for each participant.

**Fig. 2.**
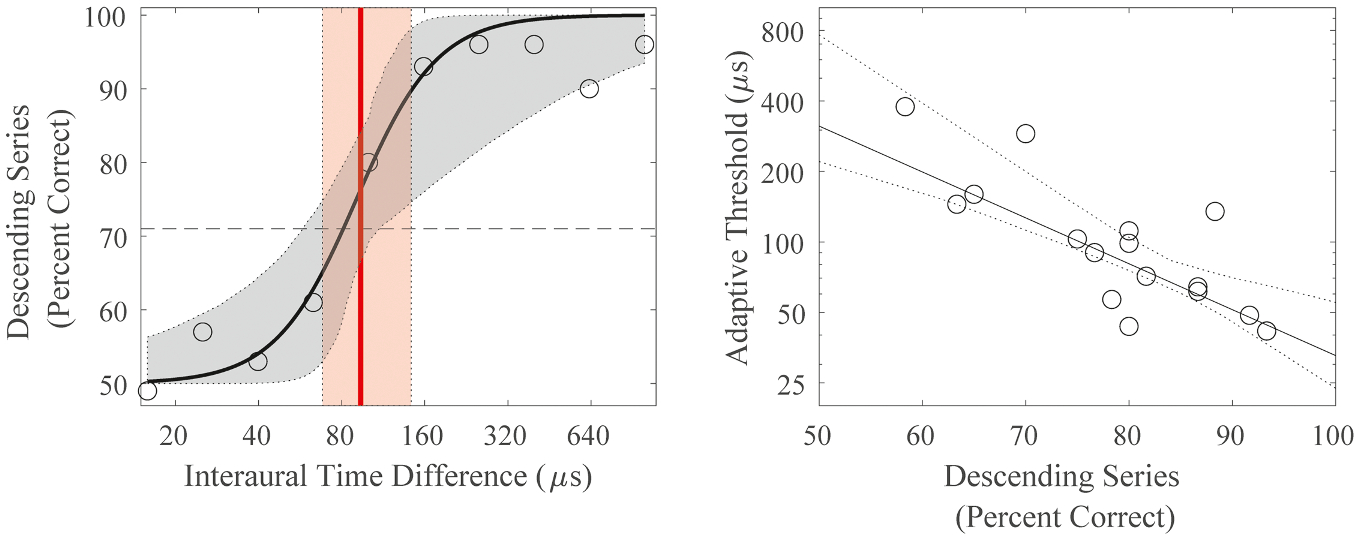
The left panel shows the relation between percent correct and ITD obtained with the descending series procedure. Each circle indicates the 20% trimmed mean percent correct for a given ITD. The data were fit with a sigmoidal function. The dashed horizontal line indicates 70.7%. The red vertical line indicates the 20% trimmed mean of the adaptive procedure threshold. The shaded areas indicate the 95% confidence intervals for the adaptive procedure threshold (light red) or for the sigmoidal function fit for the descending series procedure (grey). The right panel shows the relation between the 16 individual percent correct scores from the descending series procedure and the corresponding threshold-ITD from the adaptive procedure. Each circle indicates an individual participant. The fit, based on a least trimmed squares regression is shown. The 95% confidence intervals for that fit, based on a bootstrap analysis, are shown as dotted lines.

To compare estimated thresholds across the procedures, thresholds were first estimated for the descending series procedure. This was accomplished by fitting a logistic function, constrained to have a lower limit of 50%, to the obtained values of percent correct versus the ITD, pooled across participants (Fig. 2, left panel). The value of ITD at which the logistic fit crossed 70.7% correct was compared to the adaptive threshold, which also targeted 70.7% correct. The trimmed mean threshold-ITDs were 82 and 95 μs, for the descending series and adaptive procedures, respectively. These were compared via a percentile bootstrap pairwise comparison as follows: The participants were selected with replacement. Two bootstrap distributions were created, one for the descending series procedure and one for the adaptive procedure, each containing data from the corresponding dataset for the same participant (i.e., the dependency of the data was maintained). The difference between the threshold-ITD values derived from the two procedures did not differ significantly (95% confidence of the difference score: −12 to 49 μs).

Because it may not be possible to estimate the psychometric function consistently and accurately based on the limited number of trials used in the descending series procedure, particularly for an individual participant, it is important to determine whether threshold-ITD can be estimated accurately from the percent scores, a process carried out successfully by Gallun et al. (2013). Those investigators used a descending series procedure to estimate magnitudes of spatial release from masking. To apply that approach using our data, for *each participant*, we constructed paired values of: 1) the pooled percent correct across all values of ITD within the descending series procedure and 2) the threshold-ITD obtained in the adaptive procedure. The resulting 16 points are plotted in the right-hand panel of Fig. 2. The relation between the overall percent correct for the descending series procedure and the log of the 20% trimmed mean for the threshold for the adaptive procedure was analyzed using a bootstrap Pearson correlation. The correlation between the scores with the two procedures was significant (*r* = −0.81; 95% confidence interval: −.46 −0.94). The 16 points were also fit using a least trimmed squares regression. The fit (see Eq. (1)) is shown in the right panel of Fig. 2 and provides a means to translate a single, pooled, percent correct score obtained from the descending series to an estimate of threshold-ITD, albeit a relation tied to the specific starting ITD and step size used in the current study.

(1) $$\text{Threshold}=e^{-4.51\times \text{PercentCorrect}+8.0}$$

Variability was comparable for the two procedures. Using Eq. (1), the estimated threshold-ITD for the descending series was calculated for each run for each participant. Sn (a robust measure of standard deviation; Rousseeuw and Croux, 1993) was calculated for those five estimates of threshold-ITD for the descending series procedure (trimmed mean: 47 μs) and the five estimates of threshold-ITD for the adaptive procedure runs (trimmed mean: 52 μs). Percentile bootstrap pairwise comparisons based on 20% trimmed means indicated that these values did not differ significantly (95% confidence interval: −16.8 to 36.8 μs).

The trimmed-mean time required for the completion of a single run of each procedure was 1.8 and 3.3 min for the descending series and adaptive procedures, respectively. The differences were compared using a percentile bootstrap pairwise comparison based on 20% trimmed means. The adaptive procedure required significantly more time to complete than the descending series procedure (95% confidence for 20% trimmed mean of the time difference between tasks: 1.3 to 1.7 min). Using the first five reversals instead of the last five reversals yielded significantly more variable thresholds (20% trimmed mean of Sn was 103 μs when using the first five reversals and 52 μs when using the last five reversal; 95% confidence interval for the difference: 7.5 to 79.4 μs), suggesting that halving the number of reversals for the adaptive procedure to yield a more comparable testing time would approximately double the variability.

## 4. Discussion

Overall, the data indicate that estimated threshold-ITDs from the descending series procedure are similar to threshold-ITDs obtained from the adaptive procedure (within one step size, on average), and an empirical function relating adaptive thresholds to overall percent correct obtained in the descending procedure was determined. The use of a common data set obtained from the same group of participants across the two procedures strengthens the reliability and generalizability of the results.

A single run of the descending series procedure required significantly and substantially less time to complete than did a single run of the adaptive procedure, despite yielding comparable variability. The relatively small confidence interval for the difference in variability across procedures bolsters the reliability of this result. Halving the number of “reversals” obtained with the adaptive procedure so as to make essentially equal the time required to complete a single run of each of the two procedures resulted in about a doubling of the variability of the estimates of threshold obtained via the adaptive procedure. Thus, the same ends could not have been achieved by merely adjusting the parameters of the adaptive procedure in that manner.

Additional optimizations of the descending series procedure, such as reducing the time between stimuli, optimizing the starting ITD, or optimizing the step-size might reduce further the time necessary to obtain suitably accurate and reliable estimates of threshold-ITD. However, such adjustments could be applied to either of the procedures discussed here.

## Footnotes

1 We use the term “threshold” reluctantly. In contrast to its correct and historical meaning as a categorical barrier— a concept shown to be invalid — “threshold” has been redefined to refer to the level of the signal required to achieve a specific level of performance. We agree with Dr. Dennis McFadden that such usage does a disservice in terms of discussions about and understanding of the relevant psychophysical concepts (Hafter & McFadden, 2023).

2 ITD was decreased in dB, consistent with Bernstein & Trahiotis (2004) and Thavam & Dietz (2019), given the indication that there is a sigmoidal relationship between percent correct and the log of ITD (i.e. ITD in dB) (Saberi, 1995).
